# METTL3/miR-192-5p/SCD1 Axis Regulates Lipid Metabolism to Affect T Cell Differentiation in Asthma

**DOI:** 10.1155/mi/4955849

**Published:** 2025-01-19

**Authors:** Zhengrong Chen, Dingwei Yan, Suyu Guo, Yiyi Song, Xinxing Zhang, Wenjing Gu, Heting Dong, Li Huang

**Affiliations:** ^1^Department of Pediatric Pulmonology, Children's Hospital of Soochow University, No 303, Jingde Road, Suzhou 215003, China; ^2^Department of Pediatric Pulmonology, Xuzhou Children's Hospital, Xuzhou Medical University, No 18 Sudi Road, Xuzhou 221000, China; ^3^Suzhou Medical College, Soochow University, 199 Ren-Ai Road, Suzhou 215123, China

**Keywords:** asthma, lipid metabolism, METTL3, miR-192-5p, SCD1, T cell differentiation

## Abstract

**Background:** This study aimed to explore the mechanisms underlying T-cell differentiation in asthma.

**Methods and Results:** Flow cytometry was performed to detect Th cells. LC-MS/MS was performed to assess lipid metabolism. HE staining was performed to assess the pathological changes of the lung tissues. ELISA was performed to detect cytokine levels. The results of quantitative real-time polymerase chain reaction (qRT-PCR) and western blot showed that miR-192-5p expression was decreased, while SCD1 expression was increased in CD4^+^T cells isolated from the peripheral blood of children with asthma. The dual luciferase reporter assay determined the direct interaction between miR-192-5p and SCD1. MiR-192-5p inhibitor reduced ASCL3 and PPARα, increased FASN and SREBP1c mRNA expression and protein levels in mouse spleen CD4^+^T cells, and elevated Th2 and Th17 cells, but these effects were reversed by the SCD1 inhibitor. Oleic acid (OA) reduced Th1 cells and increased Th2 and Th17 cells in mouse spleen CD4^+^T cells treated with an SCD1 inhibitor. Additionally, pri-miR-192-5p expression was increased in CD4^+^T cells isolated from the peripheral blood of asthmatic children, and the deletion of METTL3 upregulated pri-miR-192-5p expression in an m6A-dependent manner. MiR-192-5p mimic and inhibitor both reversed miR-192-5p and SCD1 expression affected by overexpression or deletion of METTL3, both *in vivo* and *in vitro*. Furthermore, METTL3 overexpression attenuated lung inflammation, elevated Th1 cells, and reduced Th2 and Th17 cells in CD4^+^T cells isolated from the peripheral blood of asthmatic mice. These effects were reversed by the miR-192-5p inhibitor.

**Conclusion:** These results suggest that METTL3/miR-192-5p/SCD1 axis regulates lipid metabolism and affects T cell differentiation, thus affecting asthma progression. This study may provide novel insights into the pathogenesis of asthma and a new treatment strategy.

## 1. Introduction

Asthma is a common chronic airway inflammatory disease characterized by airway hyper-responsiveness, involving eosinophils, mast cells, and lymphocytes [1]. The immune response in asthma is primarily driven by CD4^+^T helper (Th) cells, which are represented by Th1, Th2, and Th17 cells, particularly Th2 cells [2, 3]. Thus, exploring the underlying pathogenesis that affects Th cells will provide novel insights into asthma treatment.

MicroRNAs (miRNAs) are small, noncoding RNA molecules that regulate gene expression at the post-transcriptional level and participate in various biological processes, including T-cell differentiation. Upregulated miR-155 induced Th1 cell differentiation by inhibiting the signal transduction pathway of IFN-*γ* in CD4^+^T cells [4]. MiR-27a and miR-214 exerted opposite regulatory roles in Th17 cell differentiation by mediating different signaling pathways in CD4^+^T cells of the peripheral blood of patients with relapsing–remitting multiple sclerosis [5]. Upregulated miR-21 [6] and miR-126 [7] promoted Th2 differentiation of CD4^+^T cells during asthma. Considering that a large number of studies have reported that miRNAs exhibited a crucial role in asthma; for example, miR-145-5p promoted asthma pathogenesis by inhibiting kinesin family member 3A expression in mouse airway epithelial cells (AECs) [8]. miR-192-5p attenuated airway remodeling by targeting MMP-16 and ATG7 in asthma [9]. miR-182-5p attenuated asthmatic airway inflammation by targeting nicotinamide adenine dinucleotide phosphate oxidase 4 (NOX4) [10]. Thus, we speculated that miRNAs may regulate CD4^+^T cell differentiation to participate in the progression of asthma.

Recently, Li et al. [11] found that pathways related to lipid metabolism were enriched in Th2 cells via single-cell transcriptomic analysis, and inhibitors of key metabolic pathways further supported the role of glucose and lipid metabolism in asthma patients. In addition, SCD1 (stearoyl-CoA desaturase 1) was highly expressed in AECs in a mouse model of house dust mite (HDM)-induced allergic airway inflammation [12]. SCD1 was a key enzyme that catalyzed the production of monounsaturated fatty acyl-CoA from saturated fatty acyl-CoA and played an important role in lipid synthesis [13, 14]. Bioinformatic analysis predicted that there were binding sites between miR-192-5p and SCD1, and the role of miR-192-5p in T cell differentiation remained unclear; thus, we hypothesized that miR-192-5p targeted SCD1 to affect lipid metabolism to regulate T cell differentiation, thus participating in the progression of asthma. In addition to the role of miR-192-5p/SCD1 in asthma, the underlying mechanisms were also explored.

## 2. Materials and Methods

### 2.1. Patients

Twenty-four subjects were recruited from the Children's Hospital of Soochow University, including 12 children with asthma and 12 healthy children. The clinical characteristics of the children with asthma are illustrated in Table S1. None of the healthy children had a history of respiratory disease. Written informed consent was obtained from all participants' parents. This study adheres to the Declaration of Helsinki and was approved by the Ethics Committee of the Children's Hospital of Soochow University (No. 2022CS184).

### 2.2. CD4^+^T Cells Isolation

Ficoll (P8900, Solarbio, Beijing, China) was used to obtain human/mouse peripheral blood mononuclear cells, and a MojoSort human/mouse CD4 T Cell Isolation Kit (BioLegend, San Diego, CA, USA) was used to obtain CD4^+^T cells from the peripheral blood mononuclear cells.

### 2.3. Cell Transfection

The shRNA targeting METTL3 and the negative control were synthesized by GenePharma (Shanghai, China). Isolated CD4^+^T cells were transfected using Lipofectamine 2000 according to the manufacturer's protocol. Transfected cells were harvested after 48–72 h.

### 2.4. Quantitative Real-Time Polymerase Chain Reaction

Human/mouse peripheral blood CD4^+^T cells and mouse spleen CD4^+^T cells were employed for the extraction of total RNA using TRIzol reagent (Invitrogen, Carlsbad, CA, USA), which were then reversely transcribed into complementary DNA using a reverse transcription kit (Tiangen Biotech, Beijing, China). Quantitative real-time polymerase chain reaction (QRT-PCR) was performed using an ABI-7500 Real Time PCR System (Applied Biosystems, Warrington, UK) with gene primers and SYBR Green PCR Master Mix (Tiangen Biotech). Data were analyzed using the 2^-∆∆Ct^ method, and β-actin or U6 was used for normalization. The primers used were as follows:

ASCL3 (forward): 5′-CAACTACAGGCGCTGTGACTACAC-3′

ASCL3 (reverse): 5′-GCTGTTCGAGGGTTCTTCTTGG-3′

Peroxisome proliferator-activated receptor α (PPARα) (forward): 5′-GTGAAGGCTGTAAGGGCTTCTTT-3′

PPARα (reverse): 5′-TTCCGAATCTTTCAGGTCGTGTT-3′

Fatty acid synthase (FASN) (forward): 5′-CAGCAAGTGTCCACCAACAAGCG-3′

FASN (reverse): 5′-TGATGCCGTCAGGTTTCAGTCCC-3′

Sterol regulatory element-binding protein 1c (SREBP1c) (forward): 5′-CACTTCTGGAGACATCGCAAAC-3′

SREBP1c (reverse): 5′-TGGTAGACAACAGCCGCATC-3′

SCD1 (forward): 5′-ACTACCACCACACCTTCC-3′

SCD1 (reverse): 5′-GTCTCCAGTTCTCTTAATCCT-3′

Methyltransferase like 3 (METTL3) (forward): 5′-CTGTTTCCATCCGTCTTGCCATCT-3′

METTL3 (reverse): 5′-AAATCCTGTTCTGCACGCCGTTT-3′

MiR-192-5p (forward): 5′-ACACTCCAGCTGGGCTGACCTATGAATTG-3′

MiR-192-5p (reverse): 5′-TGGTGTCGTGGAGTCG-3′

Pri-miR-192-5p (forward): 5′-ACACTCCAGCTGGGCGTGCACAGGGCTCTGACCTATGAAT-3′

Pri-miR-192-5p (reverse): 5′- CTCAACTGGTGTCGTGGAGTCGGCAATTCAGTTGAGGGTGAAC-3′

U6 (forward): 5′-CTCGCTTCGGCAGCACA-3′

U6 (reverse): 5′-AACGCTTCACGAATTTGCGT-3′

β-actin (forward): 5′-GGCTGTATTCCCCTCCATCG -3′

β-actin (reverse): 5′-CCAGTTGGTAACAATGCCATGT-3′

### 2.5. Western Blot Analysis

Mouse peripheral blood and spleen CD4^+^T cells were lysed, and the protein content was determined using a bicinchoninic acid assay (BCA) protein assay kit (Biosharp, Guangzhou, China). Then, the proteins were separated, transferred to PVDF membranes, and maintained with 5% non-fat milk for 1 h at room temperature. The membranes were then incubated with primary antibodies specific for SCD1 (28678-1-AP, 1 : 8000, Proteintech, Chicago, IL, USA), METTL3 (15073-1-AP, 1 : 2000, Proteintech), FASN (10624-2-AP, 1 : 20000, Proteintech), PPARα (66826-1-Ig, 1 : 2000, Proteintech), SREBP1c (66875-1-Ig, 1 : 2000, Proteintech), ASCL3 (PA5-142662, 0.1 µg/mL, Thermo Fisher Scientific), and β-actin (GB11001, 1 : 2500, Servicebio) and secondary HRP-conjugated goat anti-mouse IgG (SA00001-1-A, 1 : 3000, Proteintech). Finally, an enhanced chemiluminescence kit (Vazyme, Nanjing, China) was used to determine the protein bands, and optical density was analyzed using Image-Pro Plus 6.0. β-actin was used as an internal control.

### 2.6. Bioinformatics Analysis

Based on TargetScan (https://www.targetscan.org/vert_71/), the binding sites between miR-192-5p and SCD1 were predicted.

### 2.7. Dual Luciferase Reporter Assay

The SCD1-3′UTR-containing wild-type or mutant binding sites was cloned into the psiCHECKTM-2 vector. HEK-293 T cells were cotransfected with the miR-192-5p mimic, and mimic NC was used as control. The cells were harvested and washed twice with PBS. Finally, luciferase activity was measured using a dual-luciferase reporter assay system (Promega, Beijing, China).

### 2.8. Flow Cytometry

According to the kit protocol, for the entry of BV421 Rat antimouse IL-4 (562915, BD), BV605 Rat antimouse IL-17A (564169, BD), PE Rat antimouse IFN-γ (554412, BD), BU421 Rat antimouse CD25 (562606, BD), and PE anti-Mo/Rt FoxP3 (554412, Invitrogen), the cell membrane of mouse peripheral blood and spleen CD4^+^T cells was permeabilized by permeabilization buffer and fixed with fixation buffer. All samples were analyzed using a BD FACSCalibur flow cytometer, and the results were analyzed using FlowJo Software (Tree Star, Ashland, OR, USA).

### 2.9. Enzyme-Linked Immunosorbent Assay (ELISA)

The levels of IL-4, IFN-γ, and oleic acid (OA) in the bronchoalveolar lavage fluid (BALF), and serum were measured using a Mouse IL-4 ELISA Kit (E-EL-M0043c, Elabscience, USA), Mouse IFN-γ ELISA Kit (E-EL-M0048c, Elabscience), and Mouse OA ELISA Kit (RJ29333, Ren Jie Biotechnology, Shanghai) according to the manufacturer's protocol.

### 2.10. RNA Immunoprecipitation

Immunoprecipitation was performed using anti-m6A antibody (ab208577, Abcam) or isotype control IgG (Abcam). The coprecipitated RNA was extracted and subjected to qRT-PCR, which was normalized to the input.

### 2.11. Liquid Chromatography Tandem-Mass Spectrometry (LC-MS/MS)

Mouse spleen CD4^+^T cells transfected with inhibitor NC or miR-192-5p inhibitor were extracted for lipids and then separated using a Nexera X2 LC-30AD ultra-high-pressure liquid chromatography system (Shimadzu). A QTRAP 5500 mass spectrometer (AB SCIEX) was used to perform mass spectrometry in positive and negative ion mode. MultiQuant software was used to extract the chromatographic peak area and retention time, and the lipids were identified and quantified.

### 2.12. The Establishment of Asthma Model and Experimental Group

BALB/c mice (18–22 g) that were used as experimental animals were purchased from Charles River (Beijing, China). All experiments adhered to ARRIVE guidelines, and animal protocols were approved by the Committee of Animal Use and Care of Soochow University. To establish the asthma model, mice were intraperitoneally injected with 0.2 mL of normal saline containing 50 μg of ovalbumin (OVA, Aladdin, Shanghai, China) and 4 mg of aluminum hydroxide on days 1, 7, and 14. Starting on day 25, an airway challenge was performed daily for 30 min with aerosolized 2% OVA for 3 weeks. Mice treated with the same volume of normal saline without OVA served as controls. To examine the effect of METTL3 and miR-192-5p on asthma, the model mice were administered miR-192-5p inhibitor or oe-METTL3 via the tail vein every 7 days starting on day 3. Finally, the mice were sacrificed by intraperitoneal injection with pentobarbital (200 mg/kg), then BALF and lung tissues were collected for subsequent analysis.

### 2.13. Hematoxylin-Eosin (HE) Staining

After the fixation with 4% polyformaldehyde liquid, dehydration, and transparency, the lung tissues were embedded in paraffin and cut into 5-μm sections. The sections were then stained with hematoxylin (Solarbio) and eosin (Sangon, Shanghai, China). Images were captured under a microscope, and pathological changes in the lung tissues and infiltration of inflammatory cells were observed.

### 2.14. Statistical Analysis

Statistical analyses were performed using GraphPad Prism 7.0 (GraphPad Software, La Jolla, CA, USA), and all values were exhibited as mean ± standard deviation (SD). The differences between groups were analyzed using unpaired Student's t-test or one-way analysis of variance (ANOVA), which was followed by Tukey's post hoc test. *P* was set at *p*  < 0.05.

## 3. Results

### 3.1. MiR-192-5p Regulates Lipid Metabolism via Targeting SCD1

**Figure 1:**
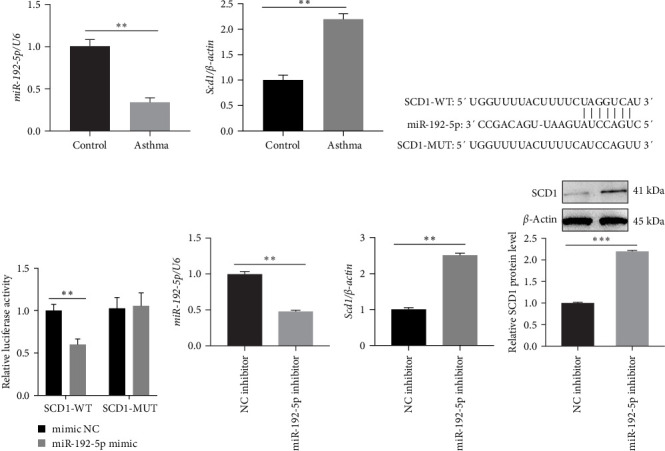
MiR-192-5p-reduced SCD1 expression via targeting SCD1. CD4^+^T cells were isolated from the peripheral blood of children with asthma and healthy children (*n* = 12), and qRT-PCR was performed to examine miR-192-5p (A) and SCD1 (B) expression in CD4^+^T cells; (C) the predicted binding sites between miR-192-5p and SCD1; (D) the dual luciferase reporter assay was employed to assess the interaction between miR-192-5p and SCD1 in HEK-293 T cells. Mouse spleen CD4^+^T cells were isolated and transfected with miR-192-5p inhibitor, qRT-PCR, and western blotting were performed to examine miR-192-5p expression (E), SCD1 mRNA expression (F), and protein level (G). *⁣*^*∗∗*^*p*  < 0.01, *⁣*^*∗∗∗*^*p*  < 0.001. All experiments were repeated for three times, and all values were exhibited as mean ± SD. The differences between groups were analyzed using unpaired Student's *t*-test and followed by Tukey's post hoc test.

**Figure 2:**
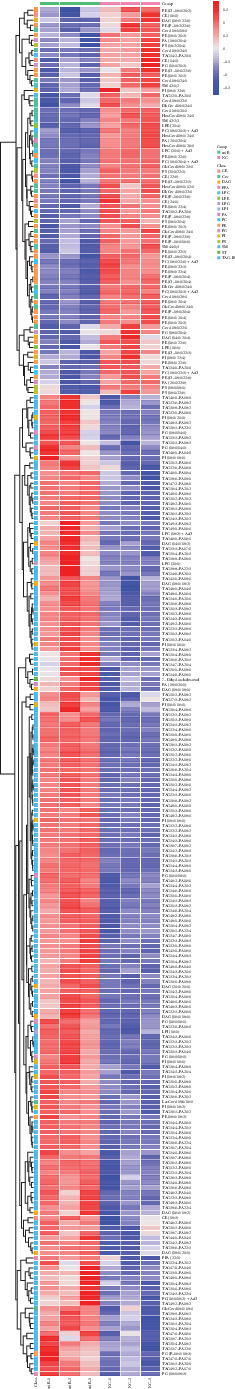
The heat map of lipid metabolism. Mouse spleen CD4^+^T cells were isolated and transfected with an miR-192-5p inhibitor, and LC-MS/MS was performed to assess lipid metabolism.

**Figure 3:**
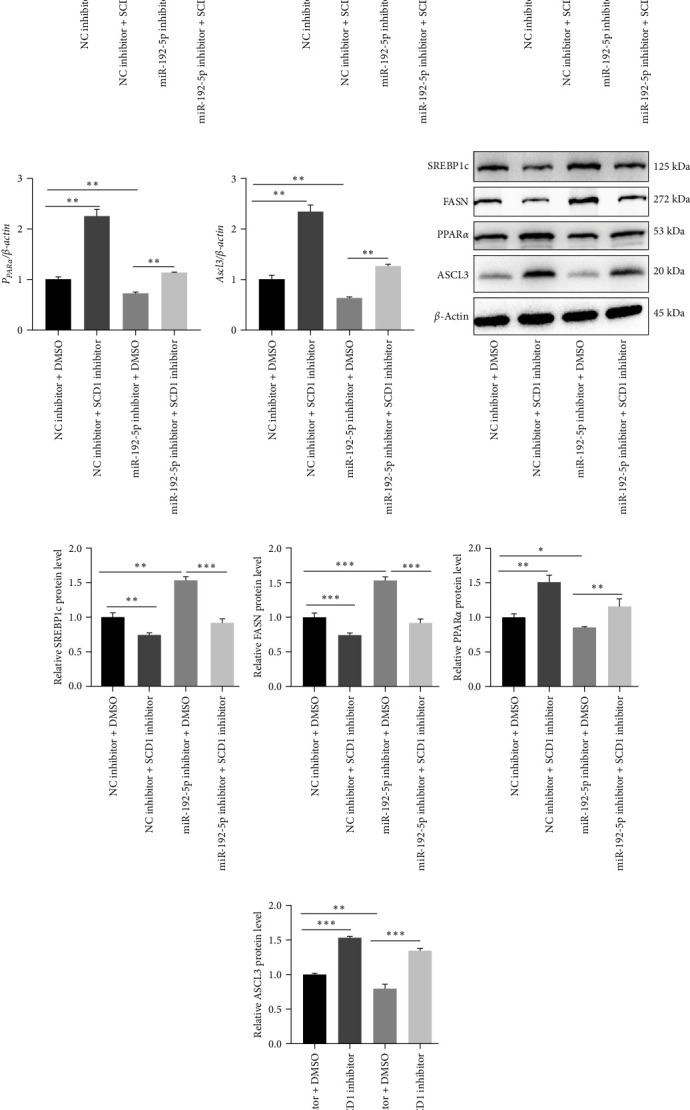
MiR-192-5p regulated lipid metabolism via targeting SCD1. Mouse spleen CD4^+^T cells were isolated and transfected with miR-192-5p or SCD1 inhibitors. qRT-PCR (A–E) and western blotting (F–J) were performed to examine miR-192-5p, ASCL3, PPAR*α*, FASN, and SREBP1c expression levels. All experiments were repeated for three times, and all values were exhibited as mean ± SD. The differences between groups were analyzed using ANOVA and followed by Tukey's post hoc test. *⁣*^*∗*^*p*  < 0.05, *⁣*^*∗∗*^*p*  < 0.01, *⁣*^*∗∗∗*^*p*  < 0.001.

We first found that mature miR-192-5p (Figure 1A) was decreased, whereas SCD1 (Figure 1B) was increased in CD4^+^T cells isolated from the peripheral blood of asthmatic children. The predicted specific binding sites between miR-192-5p and SCD1 are shown in Figure 1C, and the dual-luciferase reporter assay further confirmed the interaction between miR-192-5p and SCD1 (Figure 1D). Next, normal mouse spleen CD4^+^T cells were employed for the following *in vitro* experiments. MiR-192-5p inhibitor significantly inhibited miR-192-5p expression (Figure 1E) and increased SCD1 mRNA expression (Figure 1F) and protein level (Figure 1G) in mouse spleen CD4^+^T cells. SCD1 catalyzed the synthesis of monounsaturated fatty acids (MUFAs), mainly OA (C18 : 1) and palmitic acid (C16 : 1). LC-MS/MS revealed that mouse spleen CD4^+^T cells treated with the miR-192-5p inhibitor exhibited higher level of FA 18 : 1 and FA 16 : 1 (Figure 2). PPAR*α*, ASCL3, SREBP1c, and FASN are closely associated with lipid metabolism. QRT-PCR results firstly demonstrated that miR-192-5p inhibitor markedly reduced miR-192-5p expression in mouse spleen CD4^+^T cells (Figure 3A). Additionally, miR-192-5p inhibitor significantly increased mRNA expression of SREBP1c (Figure 3B) and FASN (Figure 3C), while reduced mRNA expression of PPAR*α* (Figure 3D) and ASCL3 (Figure 3E) in mouse spleen CD4^+^T cells, but which were all markedly reversed by SCD1 inhibitor, and western blot analysis revealed similar results in mouse spleen CD4^+^T cells (Figure 3F–J). These results suggested that miR-192-5p regulated lipid metabolism by targeting SCD1 in mouse spleen CD4^+^T cells.

### 3.2. MiR-192-5p/SCD1 Axis Regulates T Cell Differentiation via Lipid Metabolism

**Figure 4:**
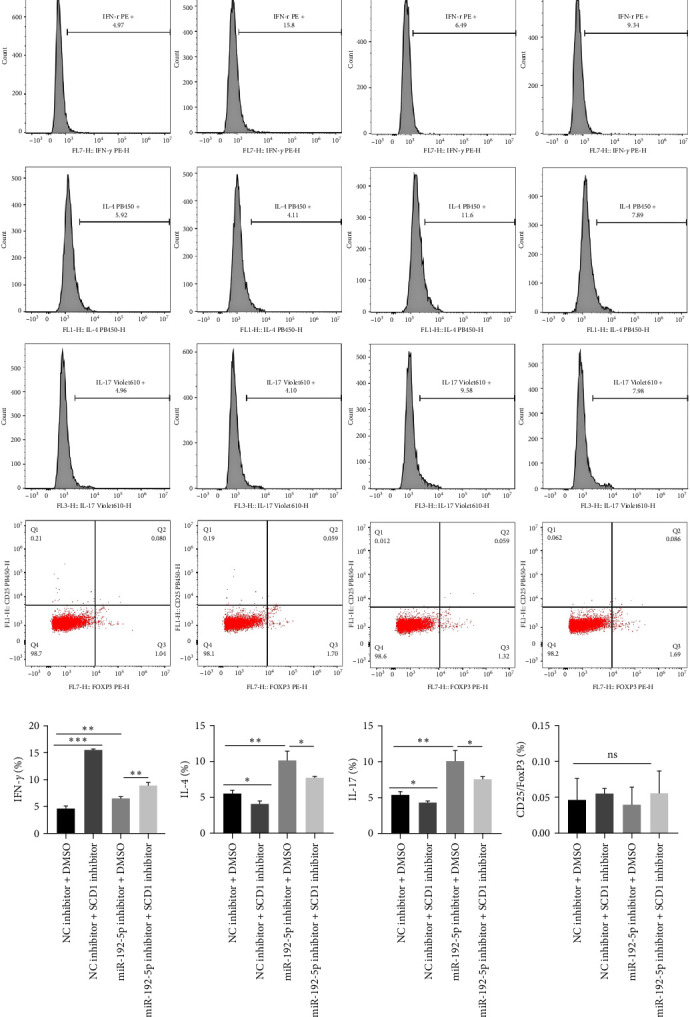
MiR-192-5p/SCD1 axis regulated T cell differentiation. Mouse spleen CD4^+^T cells were isolated and transfected with miR-192-5p inhibitor or SCD1 inhibitor, and flow cytometry was employed to detect Th1, Th2, Th17, and Treg cells (A–E). All experiments were repeated for three times, and all values were exhibited as mean ± SD. The differences between groups were analyzed using ANOVA and followed by Tukey's post hoc test. *⁣*^*∗*^*p*  < 0.05, *⁣*^*∗∗*^*p*  < 0.01, *⁣*^*∗∗∗*^*p*  < 0.001.

**Figure 5:**
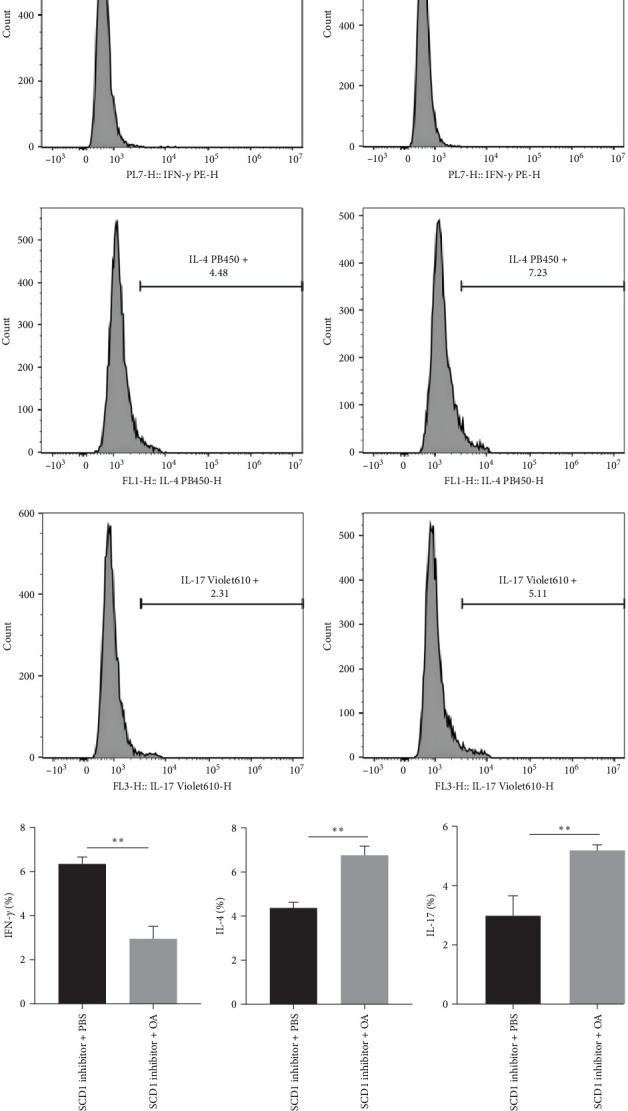
Lipid metabolism affected T cell differentiation. Mouse spleen CD4^+^T cells were transfected with SCD1 inhibitor and oleic acid (OA) was added. Flow cytometry was used to detect Th1, Th2, and Th17 cells (A–D). All experiments were repeated for three times, and all values were exhibited as mean ± SD. The differences between groups were analyzed using the unpaired Student's *t*-test, and followed by Tukey's post hoc test. *⁣*^*∗∗*^*p*  < 0.01.

Additionally, flow cytometric results revealed that increased Th1 cells induced by the miR-192-5p inhibitor were further upregulated by the SCD1 inhibitor (Figure 4A,B) in mouse spleen CD4^+^T cells, while increased Th2 and Th17 cells induced by the miR-192-5p inhibitor were markedly reversed by the SCD1 inhibitor (Figure 4A,C,D). In addition, no significant changes were observed in mouse spleen CD4^+^T cells transfected with miR-192-5p inhibitor or SCD1 inhibitor (Figure 4A,E). Furthermore, considering that miR-192-5p inhibitor exhibited more FA 18 : 1 (Figure 2) and that SCD1 catalyzed the synthesis of FA, OA was added in mouse spleen CD4^+^T cells transfected with SCD1 inhibitor. OA significantly decreased the number of Th1 cells (Figure 5A,B) and increased the number of Th2 and Th17 cells (Figure 5A,C,D). The above results suggested the regulation of Th cells by the miR-192-5p/SCD1 axis via lipid metabolism in mouse spleen CD4^+^T cells.

### 3.3. Sh-METTL3 Inhibits the Processing of Pri-miR-192-5p in an m6A-Dependent Manner to Reduce miR-192-5p Expression

**Figure 6:**
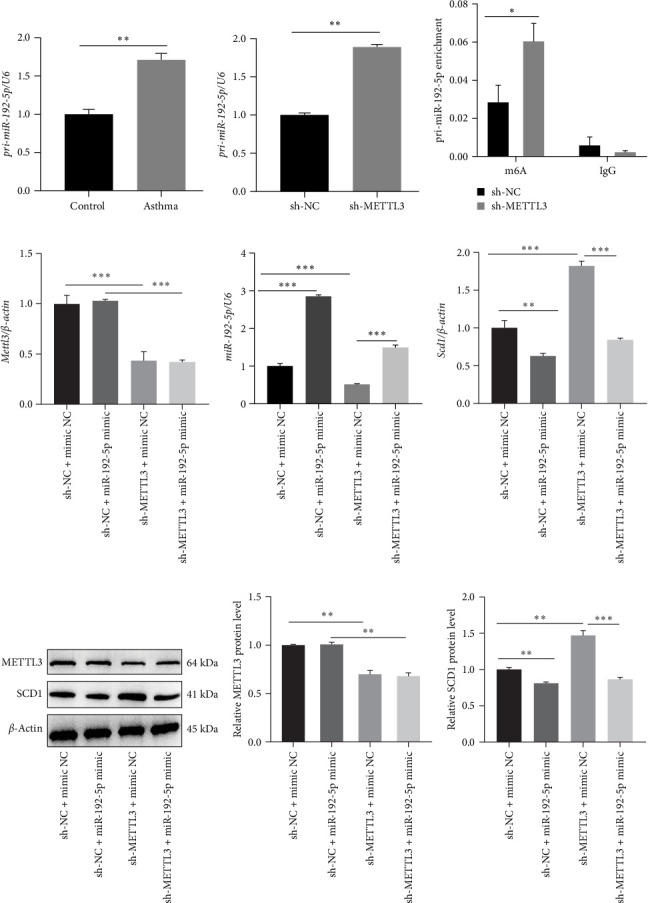
Sh-METTL3 inhibited the processing of pri-miR-192-5p in an m6A-dependent manner to reduce miR-192-5p expression *in vitro*. CD4^+^T cells were isolated from the peripheral blood of children with asthma and healthy children. qRT-PCR (A) was performed to examine pri-miR-192-5p expression in CD4^+^T cells. The mouse spleen CD4^+^T cells were transfected with sh-METTL3, qRT-PCR (B), and RIP (C) to examine pri-miR-192-5p expression. Mouse spleen CD4^+^T cells transfected with sh-METTL3 were treated with miR-192-5p mimic, qRT-PCR (D–F), and western blotting (G–I) were performed to examine METTL3, miR-192-5p, and SCD1 expression. All experiments were repeated for three times, and all values were exhibited as mean ± SD. The differences between groups were analyzed using ANOVA and followed by Tukey's post hoc test. *⁣*^*∗*^*p*  < 0.05, *⁣*^*∗∗*^*p*  < 0.01, *⁣*^*∗∗∗*^*p*  < 0.001.

Our preliminary experiments showed that METTL3 was significantly upregulated in the peripheral blood of asthmatic children and that METTL3 promoted the formation of mature miRNA by inducing the processing of N6-methyladenosine (m6A) modification-mediated miRNA precursors [15]. Therefore, we first examined the expression of pri-miR-192-5p in asthmatic children. We found that pri-miR-192-5p was increased in CD4^+^T cells isolated from the peripheral blood of asthmatic children (Figure 6A). In addition, pri-miR-192-5p increased in mouse spleen CD4^+^T cells transfected with sh-METTL3 (Figure 6B), and RNA Immunoprecipitation (RIP) experiments further showed that sh-METTL3 upregulated pri-miR-192-5p expression in an m6A-dependent manner (Figure 6C). Furthermore, qRT-PCR and western blot results revealed that sh-METTL3 significantly reduced the expression of METTL3 and miR-192-5p and increased SCD1 expression in mouse spleen CD4^+^T cells, while which were all reversed by miR-192-5p mimic (Figure 6D–I). These results suggested that sh-METTL3 reduced mature miR-192-5p expression by inhibiting the processing of pri-miR-192-5p in an m6A-dependent manner, thereby upregulating SCD1 expression in mouse spleen CD4^+^T cells.

### 3.4. METTL3/miR-192–5 p/SCD1 Axis Affects Lung Inflammation and T Cell Differentiation in Vivo

**Figure 7:**
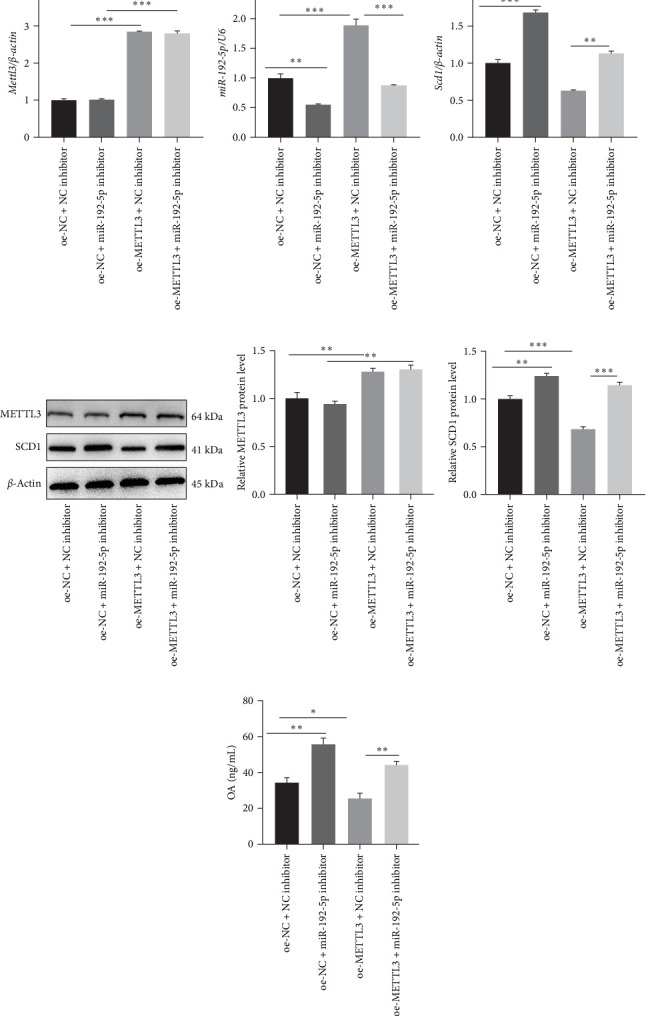
METTL3/miR-192-5p regulated SCD1 expression *in vivo*. The asthma model was established (*n* = 6), and the model mice were administered with miR-192-5p inhibitor or oe-METTL3 via the tail vein. CD4^+^T cells were isolated from the peripheral blood of the mice, qRT-PCR (A–C) and western blotting (D–F) were performed to examine METTL3, miR-192-5p, and SCD1 expression, and (G) ELISA was performed to detect serum OA levels. All experiments were repeated for three times, and all values were exhibited as mean ± SD. The differences between groups were analyzed using ANOVA and followed by Tukey's post hoc test. *⁣*^*∗*^*p*  < 0.05, *⁣*^*∗∗*^*p*  < 0.01, *⁣*^*∗∗∗*^*p*  < 0.001.

**Figure 8:**
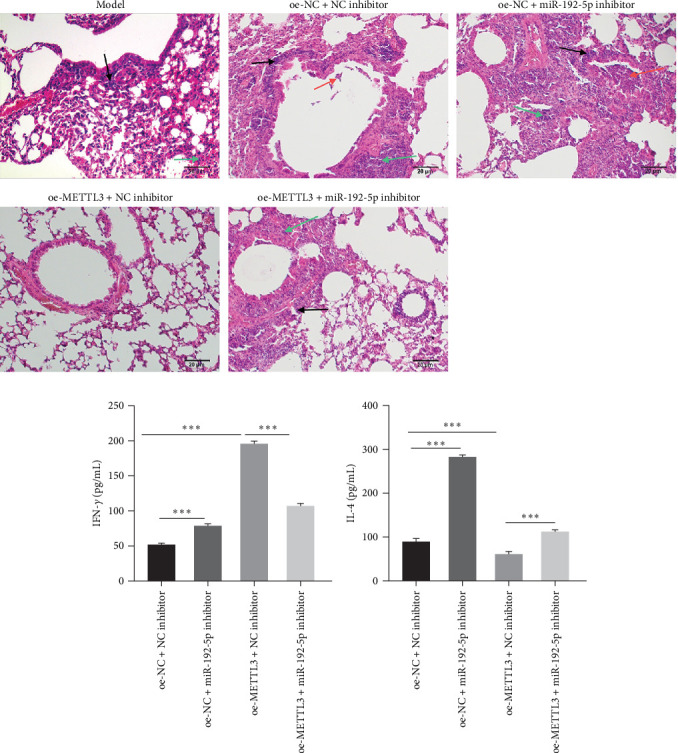
METTL3/miR-192-5p/SCD1 axis affected lung inflammation. The model mice were administered with miR-192-5p inhibitor or oe-METTL3 via the tail vein, and BALF and lung tissues were collected (*n* = 6). (A) HE staining was performed to assess the pathological morphological changes in lung tissues. ELISA was performed to detect IFN-*γ* (B) and IL-4 (C) levels in the BALF. All experiments were repeated for three times, and all values were exhibited as mean ± SD. The differences between groups were analyzed using ANOVA and followed by Tukey's post hoc test. *⁣*^*∗∗∗*^*p*  < 0.001.

**Figure 9:**
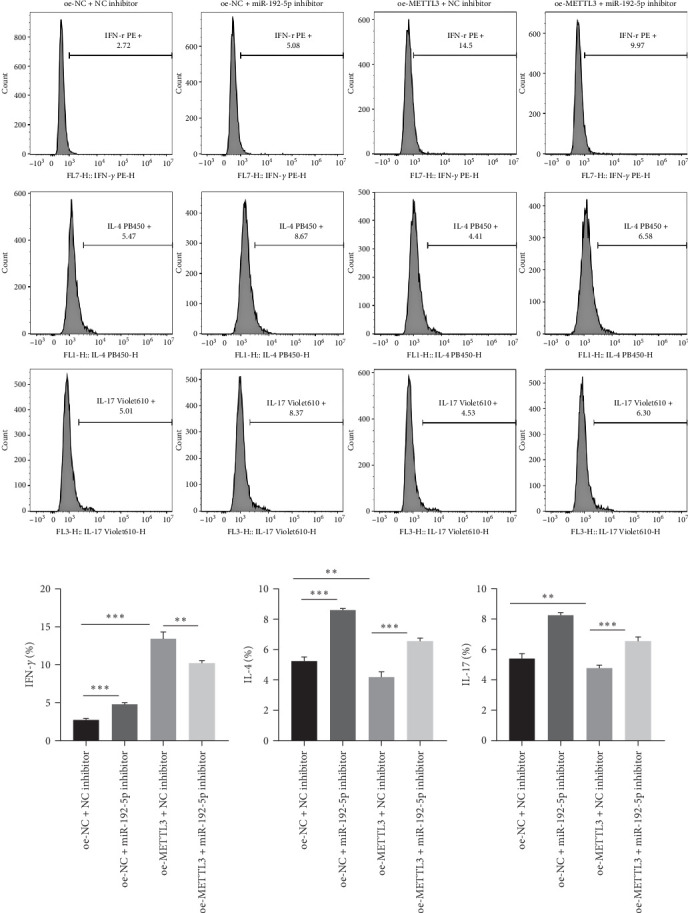
METTL3/miR-192-5p/SCD1 axis regulated T cell differentiation *in vivo*. The model mice were administered the miR-192-5p inhibitor or oe-METTL3 via the tail vein, and CD4^+^T cells were isolated from the peripheral blood of the mice (*n* = 6). Flow cytometry was used to detect Th1, Th2, and Th17 cells (A–D). All experiments were repeated for three times, and all values were exhibited as mean ± SD. The differences between groups were analyzed using ANOVA and followed by Tukey's post hoc test. *⁣*^*∗∗*^*p*  < 0.01, *⁣*^*∗∗∗*^*p*  < 0.001.

Finally, the role of the METTL3/miR-192-5p/SCD1 axis was explored *in vivo*. The asthma model mice were administered miR-192-5p inhibitor or oe-METTL3 via the tail vein. As expected, oe-METTL3 significantly increased the expression of METTL3 and miR-192-5p and reduced SCD1 expression in CD4^+^T cells isolated from the peripheral blood of asthmatic mice, which was reversed by miR-192-5p inhibitor (Figure 7A– F). In addition, the miR-192-5p inhibitor reversed the reduced OA level induced by oe-METTL3 in the serum of asthmatic mice (Figure 7G). HE staining demonstrated that the oe-NC + NC inhibitor group exhibited a large number of infiltrated inflammatory cells around the bronchus (black arrow), partial detachment of AECs (red arrow), and destruction, disappearance, and fusion of alveolar walls at the focal point (green arrow) (Figure 8A). Compared to the oe-NC + NC inhibitor group, the oe-NC + miR-192-5p inhibitor group exhibited more serious pathological morphological changes, whereas the oe-METTL3+NC inhibitor group exhibited no obvious inflammatory cell infiltration (Figure 8A). However, the attenuated pathological morphological changes in the oe-METTL3+NC inhibitor group were markedly reversed in the oe-METTL3+ miR-192-5p inhibitor group (Figure 8A). ELISA results showed that the oe-METTL3+NC inhibitor group exhibited the elevated level of IFN-*γ* (Figure 8B) and reduced IL-4 (Figure 8C) in BALF in comparison to the oe-NC + NC inhibitor group, whereas these were both reversed in the oe-METTL3+miR-192-5p inhibitor group. Flow cytometry results revealed that, compared to the oe-NC + NC inhibitor group, the oe-METTL3+NC inhibitor group exhibited elevated Th1 cells (Figure 9A,B) and reduced Th2 (Figure 9A,C) and Th17 cells (Figure 9A,D) in the peripheral blood CD4^+^T cells, while which were all markedly reversed in the oe-METTL3+miR-192-5p inhibitor group (Figure 9A–D). These results indicated that METTL3/miR-192-5p/SCD1 axis affected lung inflammation and T cell differentiation in asthmatic mice.

## 4. Discussion

Increasing evidence has shown that lipid metabolism plays a crucial role in asthma, and the abnormal lipid metabolism induces the production of inflammatory cytokines and promotes the occurrence of asthma [16, 17]. Considering that Th2 cells played a pathogenic role in asthma, and that the pathways related to lipid metabolism were enriched in Th2 cells [11], thus, in this study, we focused on the role of lipid metabolism in CD4^+^T cells associated with asthma [18]. SCD1 was a key enzyme that played an important role in lipid synthesis [13, 14], which was highly expressed in AECs in a mouse model of HDM-induced allergic airway inflammation [12]. However, the specific role of SCD1 in asthma and the underlying mechanisms were unclear.

In this study, we found that SCD1 was significantly increased in CD4^+^T cells isolated from the peripheral blood of asthmatic children, and the dual luciferase reporter assay confirmed that miR-192-5p targeted SCD1. It has been reported that miR-192-5p attenuated airway remodeling in asthma [9]; thus, we speculated that miR-192-5p may target SCD1 to affect lipid metabolism, thus affecting asthma. PPAR*α* and SREBP1c were key transcription factors that modulated lipid synthesis [19]. Alcohol exposure promoted hepatic fatty acid synthesis by activating SREBP1c, which affected de novo lipogenesis via upregulating lipogenic enzymes, including acetyl-CoA carboxylase 1 (ACC1) and FASN [20]. Also, Cao et al. [21] reported that SCD1 overexpression upregulated the expression of SREBP1c and FASN while downregulated the expression of PPAR*α* and ASCL3 in HepG2 cells. As expected, our results demonstrated that miR-192-5p inhibitor reduced the expression of ASCL3 and PPAR*α*, while increasing the expression of FASN and SREBP1c, but these were all markedly reversed by SCD1 inhibitor, suggesting the regulation of lipid metabolism by miR-192-5p/SCD1 axis in CD4^+^T cells in asthma.

N6-methyladenosine (m6A) was a type of RNA modification that was regulated by the dynamic interactions between m6A writers (such as methyltransferase-like (METTL)3, METTL14 and Wilm tumour1-associated protein (WTAP) [22, 23]), erasers (such as fat mass and obesity-associated protein (FTO) and AlkB homolog 5 (ALKBH5) [24, 25]), and reader proteins (such as YT521-B homology (YTH) and insulin-like growth factor-binding proteins (IGFBPs) [26]). M6A played a key role in various pathophysiological processes. METTL14 inhibited hepatocellular carcinoma metastasis by inducing miR-126 mature [27]. METTL3 facilitated prostate cancer progression by upregulating LEF1 m6A methylation [28]. FTO induced cell transformation by proto-oncogenes and resulted in acute myeloid leukemia [29]. Our preliminary experiments have revealed that METTL3 was significantly upregulated in the peripheral blood of asthmatic children, suggesting a potential role of METTL3 in asthma. METTL3 induced the formation of mature miRNAs by promoting the processing of m6A modification-mediated miRNA precursors [15]. In addition, m6A modification was associated with T cell homeostasis and Th cell disorders [30]. In this study, we found that pri-miR-192-5p was increased in CD4^+^T cells isolated from the peripheral blood of asthmatic children and sh-METTL3 reduced mature miR-192-5p expression by inhibiting the processing of pri-miR-192-5p in an m6A-dependent manner; thus, we speculated that METTL3 promoted the processing of pri-miR-192-5p into mature miR-192-5p to target SCD1 in CD4^+^T cells in asthma. To our delight, both the *in vivo* and *in vitro* experiments revealed that the regulation of SCD1 expression by METTL3 was reversed by miR-192-5p in CD4^+^T cells. The *in vivo* experiments also revealed that the promotion effect of METTL3 on lung inflammation was reversed by miR-192-5p inhibitor in asthmatic mice.

The differentiation disorders of Th1, Th2, Th17, and Treg cells were also closely associated with asthma [31]. Perucha et al. [32] reported that Th1 cells treated with statins switched from a pro-inflammatory (IFN-*γ*^+^) to anti-inflammatory (IL-10^+^) phenotype, and the cholesterol biosynthesis pathway was blocked in rheumatoid arthritis (RA), indicating that cholesterol biosynthesis was required for the pro-inflammatory function of Th1 cells. The inhibition of SCDs in a mouse model resulted in Th cell apoptosis, indicating that fatty acids were critical for Th cell survival [33]. In this study, we observed that mouse spleen CD4^+^T cells transfected with miR-192-5p inhibitor exhibited more FA 18 : 1 and FA 16 : 1, and OA significantly decreased Th1 cells while increasing Th2 and Th17 cells in mouse spleen CD4^+^T cells transfected with SCD1 inhibitor, suggesting the regulation of Th cells of miR-192-5p/SCD1 axis via lipid metabolism in CD4^+^T cells. Furthermore, the regulation of METTL3 on T cell differentiation was also reversed by miR-192-5p inhibitor in CD4^+^T cells isolated from the peripheral blood of asthmatic mice.

Based on the above results, we concluded that METTL3 promoted the processing of pri-miR-192-5p into mature miR-192-5p to target SCD1 to affect lipid metabolism, further regulating T cell differentiation and facilitating asthma development, which may provide a novel therapeutic strategy for asthma.

## Data Availability

The data that support the findings of this study are available from the corresponding author, [LH], upon reasonable request.

## Supporting Information

Supporting Information Table S1. The clinical characteristics of asthma children.
