# Supplementary material for: METTL3/miR-192-5p/SCD1 Axis Regulates Lipid Metabolism to Affect T Cell Differentiation in Asthma
Source: Mediators Inflamm. 2025 Jan 19;2025:4955849. doi: 10.1155/mi/4955849 (PMC11769594; doi:10.1155/mi/4955849)
Supplement: Supporting Information — Table S1. The clinical characteristics of asthma children. [file 4955849.f1.doc]

**Supplemental Table1 The clinical characteristics of asthma children**

| Number | Age (year) | Sex | Asthma years | Seasonal asthma | Attack degree |
| --- | --- | --- | --- | --- | --- |
| 1 | 6 | Male | 1 | No | Moderate |
| 2 | 5 | Male | 1 | No | Mild |
| 3 | 5 | Female | 1.5 | Yes | Severity |
| 4 | 10 | Male | 3 | Yes | Danger |
| 5 | 12 | Male | 4 | No | Severity |
| 6 | 4 | Female | 1.5 | No | Severity |
| 7 | 5 | Male | 1 | No | Mild |
| 8 | 9 | Female | 3.5 | Yes | Severity |
| 9 | 8 | Male | 2 | No | Moderate |
| 10 | 7 | Male | 3 | No | Danger |
| 11 | 14 | Female | 4 | Yes | Severity |
| 12 | 11 | Female | 5 | No | Moderate |
